# ERBB1- and ERBB2-Positive Medullary Thyroid Carcinoma: A Case Report

**DOI:** 10.3390/diseases6020025

**Published:** 2018-04-10

**Authors:** Michele Minuto, Emanuela Varaldo, Gianluca Marcocci, Amleto de Santanna, Ermanno Ciccone, Katia Cortese

**Affiliations:** 1 DISC, Department of Surgical Sciences, University of Genoa, Largo R. Benzi, 8, 16132 Genoa, Italy; Michele.Minuto@unige.it (M.M.); Emanuela.Varaldo@unige.it (E.V.); gianlucamarcocci91@gmail.com (G.M.); 2 DIMES, Department of Experimental Medicine, University of Genoa, Via Antonio de Toni 14, 16132 Genoa, Italy; amletodesantanna@unige.it (A.D.S.); cicc@unige.it (E.C.)

**Keywords:** MTC, calcitonin, parafollicular C cells, secretory granules, immunofluorescence, ultrastructure, transmission electron microscopy, ERBB1, ERBB2

## Abstract

Medullary thyroid carcinomas (MTCs) are rare thyroid tumors occurring in both sporadic and hereditary forms, whose pathogenesis is related to RET proto-oncogene alterations. MTCs originate from parafollicular cells, which produce calcitonin that represents the biochemical activity of MTC. Total thyroidectomy is the main treatment for MTC and often cures patients with confined diseases. In the presence of metastasis, the therapeutic approach depends on the rate of disease progression. We report a case of a 54-year-old female with a single, incidentally discovered, thyroid nodule of 1 cm, classified as suspicious MTC after a stimulation test with intravenous (iv) calcium. After surgery, we examined the nodule using immunohistochemistry, immunofluorescence, and electron microscopy. In addition to calcitonin, we found that it expressed intracellular positivity for the tyrosine kinase RTK receptors ERBB1 and ERBB2. Consistently with MTC features, the ultrastructural examination of the tumor displayed heterogeneous spindle-shaped cells containing two groups of secretory granules. Because of the significant correlation found between high ERBB1/ERBB2 levels in MTCs and extrathyroidal growth, the detection of ERBB1 and ERBB2 expression suggests that the two oncoproteins may be involved in the tumor proliferative responses and/or in the differentiation of parafollicular C-cells. The biological, prognostic, and therapeutic significance of these patterns would merit further investigations.

## 1. Introduction

Medullary thyroid cancers (MTCs) are rare neuroendocrine tumors arising from differentiated parafollicular cells (C cells) of the thyroid gland. The current understanding of C cells proposes that their precursors arise in the foregut endoderm and not in the neural crest, arguing that MTC should be reclassified to the family of neuroendocrine tumors with endodermal origin [1]. MTC accounts for less than 5% of all thyroid cancers worldwide. Its prognosis, although worse than that of differentiated thyroid cancers arising from the follicular cells, is overall favorable, with a survival of 80–85% at 5 years and 70–75 at 10 years for patients with localized disease, despite the presence of residual disease in more than 50% of patients treated with surgery [2]. The prognosis for metastatic MTC is worse, since only 20% of patients with distant metastases at diagnosis survive for 10 years [3]. MTCs secrete calcitonin and carcinoembryonic antigen (CEA), and deposits of amyloid are frequently observed [4]. Calcitonin (Ct) is the most sensitive and specific tumor marker both at the preoperative diagnosis and at the post-surgery follow up. Its values suggest the diagnosis of MTC in patients with thyroid nodules and then reflect the oncologic radicality of surgery, since Ct is not detectable after complete resection of the tumor and decreases after its debulking, revealing the presence of residual disease.

MTCs can either occur sporadically (75%) or as part of a genetic disease (e.g. multiple endocrine neoplasia (MEN) type 2 syndrome, or familial MTC). Inherited forms of MTC are due to autosomal dominant mutations of the RET proto-oncogene with incomplete penetrance, often presenting as multifocal disease in a background of C-cell hyperplasia (CCH). So far, total thyroidectomy is the only successful option for the treatment of the confined disease, whereas the clinical efficacy of RET inhibitors as targeted agents for the treatment of metastatic MTC is limited because of intrinsic and acquired resistance [5]. Therefore, additional molecular candidates as prognostic markers and/or potential therapeutic targets are needed. ERBB family members, such as epidermal growth factor receptor 1 (ERBB1), ERBB2, ERBB3, and ERBB4, are widely distributed in organ tissues, including endocrine organs [6], and they are strongly implicated in various human carcinomas [7]. A significant correlation between high ERBB1 and ERBB2 levels and extra thyroidal growth has been reported in MTCs [8,9]. In particular, ERBB2 expression, a non-autonomous amplifier of the ERBB signaling network [10], has been found especially enriched in C-cell hyperplasia areas within MTCs. Thus, to investigate the invasive potential of the presented case of MTC, we analyzed the expression levels of two ERBB family members, e.g. ERBB1 and ERBB2, and performed ultrastructural analysis.

## 2. Case Presentation

**Figure 1:**
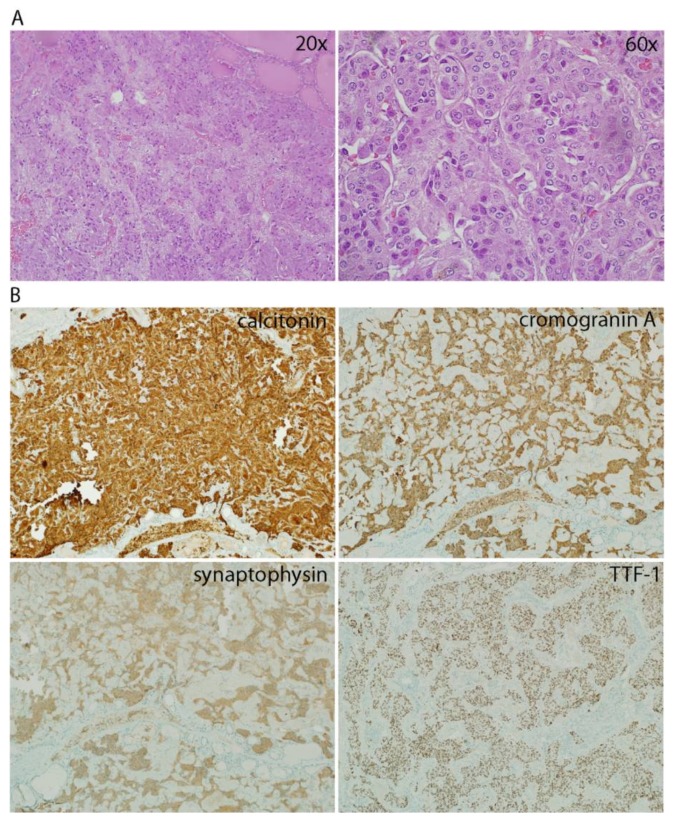
(**A**) Hematoxylin–Eosin (H&E) staining of the medullary thyroid cancer (MTC) nodule at 20× and 60× magnification. (**B**) Immunohistochemistry of the MTC nodule for calcitonin, cromogramin A, synaptophysin, and TTF-1. Magnification 10×.

We present the case of a 54-year-old female coming to our attention in April 2017 for a single thyroid nodule of 1 cm in its largest diameter, incidentally discovered in the left lobe during an ultrasound US performed for another indication. When the complete biochemical screening (TSH, autoantibodies and Ct) was performed, Ct was found only slightly elevated (40 ng/mL, normal values: 1–4.8), therefore a stimulation test with iv calcium was performed. After stimulation, Ct levels peaked at 1420 ng/mL, indicating surgical treatment. The patient underwent total thyroidectomy and central neck dissection (level VI) on the side of the tumor. The postoperative course was uneventful, with only a slight hypocalcemia recorded in the first postoperative day, which completely recovered 48 hours after surgery when the patient was discharged. Immunohistochemistry performed on the nodule showed the presence of a polilobulated medullary thyroid cancer (MTC) of 1 cm, composed of cells with fused shape with eosynophilic cytoplasm, agglomerated in solid nests with a predominantly expansive growth pattern (Figure 1A). A histological examination showed that the tumor cells were positive for Ct, Cromogranin A, Synaptofisin, and TTF-1 (Figure 1B), and negative for the presence of amyloid (not shown). Focal foci of C-cells hyperplasia were spread in the entire gland. In none of the lymph nodes of the central compartment metastases were found. 

**Figure 2:**
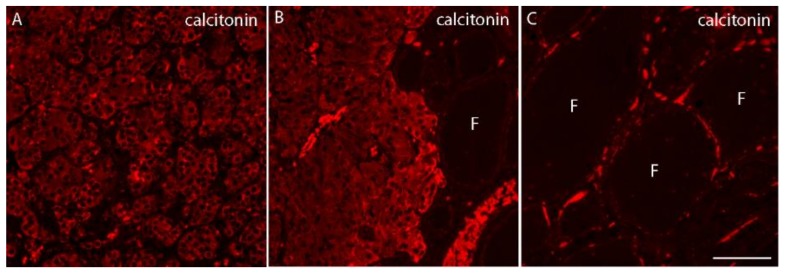
Immunofluorescence labeling for calcitonin in semi-thick sections of the MTC nodule. (**A**,**B**) represent tumor C cells positive for intracellular calcitonin. In (**C**), normal thyroid follicles (F) surrounding the MTC nodule are depicted, showing the presence of C cells positive for calcitonin. Images were taken at 40× magnification, scale bar 10 µm.

To perform a more detailed morphological analysis, formalin-fixed paraffin-embedded sections (3-μm-thick) were subjected to antigen retrieval with citrate buffer at high pH, immunolabeled with rabbit monoclonal anti-calcitonin (SP17, Cell Marque) (Figure 2), and then incubated with appropriate fluorescent secondary antibodies (anti-rabbit Alexa546) from Invitrogen/Life Technologies. As shown in Figure 2, the tumor cells as well as the C-cells found in the surrounding follicolar parenchima expressed strong intracellular positivity for Ct (Figure 2A,B), (F) (Figure 2C). 

**Figure 3:**
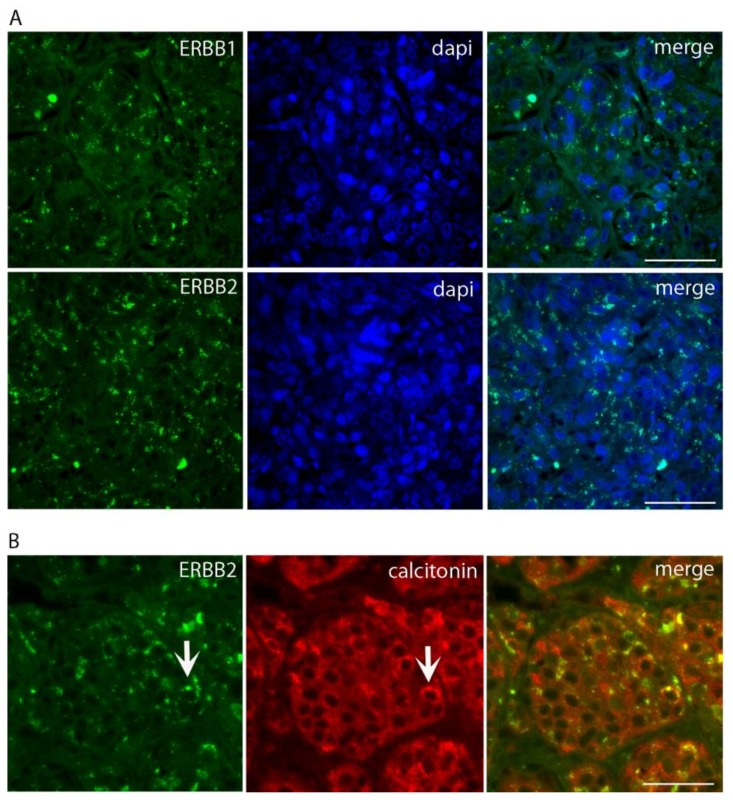
Immunofluorescence labeling for ERBB1 and ERBB2 in semi-thick sections of the MTC nodule. (**A**) represents tumor C cells positive for intracellular punctate staining for ERBB1 and ERBB2. Magnification 40×, scale bar 10 µm. In (**B**), a double immunolabeling for ERBB2 and calcitonin is depicted, showing only a partial co-localization of the two proteins (arrows). Magnification 63×, scale bar 30 µm.

We next performed immunofluorescence labeling for the RTK receptors ERBB1 (EGFR), using a polyclonal anti-EGFR antibody (Sigma-Aldrich SRL, Milano, Italy), and ERBB2, using the monoclonal humanized anti-ERBB2 antibody trastuzumab, alexa488-conjugated (10 µg/mL), (Genetech-Roche, South San Francisco, CA, USA). The slides were examined and imaged with an inverted Olympus microscope (Olympus Italia, Segrate, Italy) at 40× and 63× magnifications. As shown in Figure 3A,B, the tumor cells displayed strong punctate positivity for both ERBB1 and ERBB2 receptors, consistent with their localization in intracellular compartments, likely the endolysosomal system. Indeed, double immunofluorescence for ERBB2 and Ct showed a limited overlap of the two proteins, indicating that Ct and ERBB2 were segregated in distinct intracellular compartments. 

**Figure 4:**
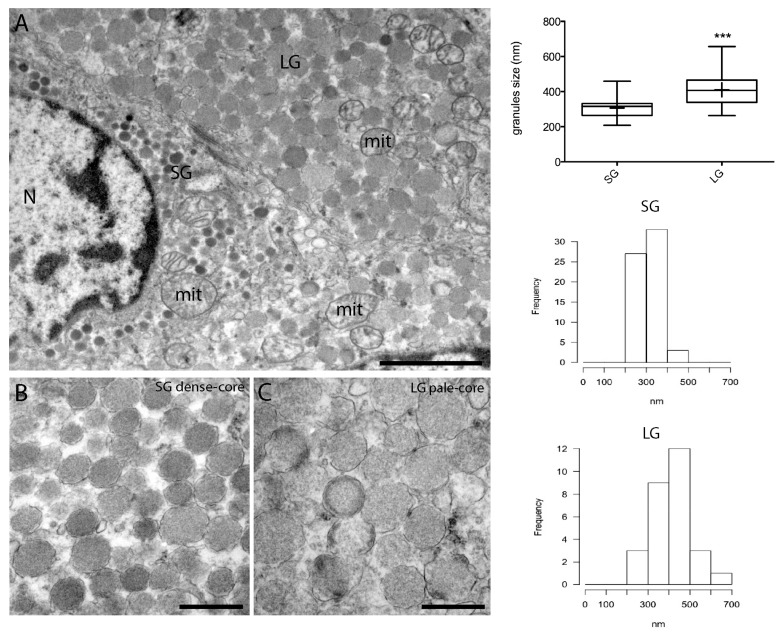
Representative TEM micrographs of the MTC nodule. (**A**) Two adiacent cells containing small dense-cored granules (SG) and large pale-cored granules (LG). Nucleus (N), mitochondria (mit). (**B**,**C**). Higher magnification insets of (**A**) showing granules morphology. Scale bars: 3 µm (**A**), 500 nm (**B**,**C**). In the right side of the panel, a box plot and a histogram representing the frequency distribution of granules’ size are shown. LG granules are significantly larger than SG granules (*** *p* < 0.0001, *t*-test).

For electron microscopy, small pieces of fresh tissue were immediately fixed after surgical resection in 2.5% glutaraldehyde, post-fixed with 1% osmium tetroxide, and embedded in epoxy resin. Ultrathin sections were double stained with uranyl acetate and lead citrate and examined under a transmission electron microscope (TEM, CM10 Philips, Eindhoven, The Netherlands). The ultrastructural examination of the tumor nodule displayed heterogeneous spindle-shaped cells containing two distinct classes of tightly packed secretory granules uniformly distributed throughout the cytoplasm (Figure 4A). 

Round-shaped mitochondria (mit) with disorganized cristae were frequently observed (Figure 4A). Tumor cells containing large, pale-cored secretory granules (LG) were the majority with respect to cells containing small and dense-cored granules (SG). The tumor nodule also contained blood and connective tissue in which cancer cells were scattered. Consistently with other morphological studies [11,12], a morphometric analysis of granule size and distribution showed two main classes of secretory granules based on their size and morphology (Figure 4B,C). The small granules (SG) had an electron-dense core and a size ranging from 200 to 400 nm, while the large granules (LG, 300–500 nm in size) were pale-cored. Statistical significant differences in granules’ size (*** *p* < 0.0001) and morphology might reflect distinct stages of hormonal production [11,12]. In particular, the mature granules appeared characterized by their uniform and moderate electron density and by their generalized distribution throughout the cytoplasm, while the small electron-dense granules would represent pre-secretory immature granules [13].

## 3. Discussion

Medullary thyroid carcinoma (MTC) is a rare calcitonin-secreting neoplasm derived from C cells that occurs in both a familial and a sporadic form [14]. It is the most aggressive among well-differentiated thyroid cancers, with survival rates of 40–80% at 10 years [15].

Because of the relative aggressiveness of MTC, the routine measurement of serum calcitonin is suggested as an integral part of the diagnostic evaluation of thyroid nodules to unmask its diagnosis [2]. When an elevated calcitonin level is found, a calcitonin stimulation test with calcium is often indicated to allow an early diagnosis and ultimately decrease the morbidity and mortality of this tumor [2,3,16]. Serum Ct is the most specific and sensitive marker of MTC, not only for its diagnosis, but also during the postsurgical follow-up [17], since it is produced in high concentrations by almost 100% of primary and metastatic MTCs. However, in a few cases, serum calcitonin can be negative or only slightly increased [18]. 

Primary MTC is efficiently controlled by surgery, but only when the tumor is confined to the thyroid [3]. Patients with progressive or symptomatic metastatic disease who cannot be treated by surgery should be considered as candidates for systemic and/or targeted therapies. Current and experimental targeted therapies for advanced medullary thyroid carcinomas include pan-tyrosine kinase (TK) inhibitors, such as sorafenib, sunitinib, and vandetanib [19,20,21,22].

The ERBB family of receptor tyrosine kinases has been implicated in carcinogenesis, with special attention to EGFR (ERBB1) and ERBB2. In particular, the expression or activation of EGFR and ERBB2 and related signaling circuitries are altered in many epithelial tumors, especially breast, gastric, lung, and ovarian cancers [10]. Both pre-clinical and clinical studies indicate that ERBB receptors have important roles in tumor progression and distant metastasis [7]. Accordingly, these receptors have been intensely studied to understand their importance in cancer biology and as therapeutic targets, and many ERBB inhibitors are now used in the clinic [23,24]. We report the case of a female patient with no familiar history of MTC presenting a single thyroid nodule of 1 cm, incidentally discovered. The nodule was classified as suspicious MTC, and the patient was admitted for total thyroidectomy. After a successful post-surgery recovery, the patient was discharged with recommendation for one-year follow up. We examined the structural and ultrastructural features of this case of MTC by immunofluorescence and electron microscopy and found that almost all tumor cells showed strong granular staining for Ct, as expected. In addition, tumor C cells displayed strong positivity for ERBB2 and ERBB1, but in distinct subcellular compartments, most likely the endolysosomal compartment. As the presence of EGFR and ERBB2 within the endocytic system is related to the propagation of pro-survival signaling originating from endosomes [13], our finding supports the hypothesis of a role for these receptors in oncogenic C cells proliferation. The ultrastructural morphology of MTC showed the presence of uniformly distributed secretory granules of different size and morphology. Tumor C cells containing large, pale-cored, secretory granules were more represented with respect to cells containing small and dense-cored granules. These characteristics suggest that MTC cells might store and elaborate precursor forms of Ct and/or produce different hormones. As this nodule was actively producing calcitonin after calcium stimulation, our morphological observation of predominant C cells containing mature, pale-cored, granules is in agreement with this hypothesis. To our knowledge, the biological and prognostic significance of ERBB expression in sporadic MTCs and in endocrine organs is still largely unknown [6]. However, ERBB1 and ERBB2 expression in MTCs has been recently correlated with more aggressive disease and extrathyroid growth [8], suggesting a role for ERBB1 and ERBB2 as important prognostic markers for tumor invasiveness. Thus, deregulation of these receptors and their signaling pathways might be connected with essential MTC properties, such as tumor cell proliferation and invasive behavior [25]. 

## 4. Conclusions

MTCs are rare but aggressive thyroid carcinomas that produce calcitonin. The therapeutic option for patients with confined MTCs is total thyroidectomy, while the treatment options for metastatic medullary thyroid carcinoma are still limited. The overexpression of ERBB receptors has been related to metastasis in various cancers, including MTCs. The presented case suggests that further preclinical and clinical studies are required to establish the role of ERBB1 and ERBB2 receptors in C cell proliferation and their potential as novel biomarkers for tumor invasiveness and as candidates for additional targeted therapies.
